# The PATCH study: Prevalence of Hearing Loss During Ageing and Treatment Choices in Osteogenesis Imperfecta: A Danish Nationwide Register-Based Cohort Study

**DOI:** 10.1007/s00223-024-01253-w

**Published:** 2024-07-16

**Authors:** Sara Kretzschmar Haumann, Jesper Roed Sørensen, Jesper Hvass Schmidt, Lars Folkestad

**Affiliations:** 1 Department of Endocrinology, Bone and Mineral Unit, Odense University Hospital, Kløvervænget 6, 5 Floor, 5000 Odense C, Denmark; 2 Department of ORL-Head and Neck Surgery and Audiology, Odense University Hospital, Odense, Denmark; 3 Department of Clinical Research, University of Southern Denmark, Odense, Denmark

**Keywords:** Osteogenesis imperfecta, Hearing loss, Stapedectomy, Stapedotomy, Hearing aids, Collagen type 1, Epidemiology

## Abstract

Osteogenesis imperfecta (OI) is a group of rare hereditary collagen disorders. Hearing loss (HL) is a known complication linked to changes in the bones of the middle ear seen in OI. We aimed to determine the prevalence, age at debut, incidence, and risk of HL, surgery on bones of the middle ear, and use of hearing aids. A Danish nationwide, register-based cohort study. Data were extracted from the Danish National Patient register. Anyone with an OI diagnosis between January 1st 1977 and December 31st 2018, matched 1:5 with a reference population (Ref.Pop) on birthyear and sex, were included. 864 persons (487 women) with OI were included in the study and 4276 (2330 women) in the Ref.Pop. The sub-hazard ratio (SHR) for any HL was 4.56 [95% CI 3.64–5.71], with a prevalence of 17.0% and 4.0% in the OI cohort and Ref.Pop. Median age at debut was 42 and 58 years, respectively. The risk of otosclerosis and/or surgery was higher in the OI cohort (SHR 22.51 [95% CI 12.62–40.14]), with a median age at debut of 43 and 32 years in the OI cohort and Ref.Pop, respectively. Hearing aid use was more frequent in the OI cohort (SHR 4.16 [95% CI 3.21–5.40]) than in the Ref.Pop. The median age at debut was 45 and 60 years in the OI cohort and Ref.Pop, respectively. Persons with OI have a higher risk and prevalence of HL, hearing aids, and surgery, debuting younger, and prevalence increases with age.

## Introduction

Osteogenesis imperfecta (OI) is a group of rare Mendelian connective tissue disorders, with a prevalence of 10.3 per 100,000 in Denmark [1]. Most OI cases are caused by an autosomal dominant mutation in the COL1A1 and COL1A2 genes, resulting in quantitative or qualitative defects in type I collagen, but mutations to genes encoding proteins required for post-translational modification, processing, or secretion of type 1 collagen, and genes important for skeletal mineralization or osteoblast function are seen [2, 3]. Type I collagen is the main component of the extracellular matrix found in bone [4]. People living with OI have decreased bone mass and hypermineralisation of the collagen fibrils, making the bone stiffer and more brittle, able to absorb less energy before breaking [5, 6]. Type I collagen is found in many connective tissues resulting in extraskeletal manifestations of OI such as dentinogenesis imperfecta, blue sclerae, cardiovascular disease, reduced lung capacity, eye disease, and hyperlaxity of ligaments [2, 3, 6–8]. OI is classified according to the revised Sillence criteria [9], that separates OI into five subtypes based on clinical features and severity ranging from few symptoms (type 1 OI) to perinatally lethal (type 2 OI).

Hearing loss can be divided into three different groups, the conductive, the sensorineural and a mixed hearing loss with components of both conductive and sensorineural hearing loss. Where the conductive hearing loss is due issues related to the small bones of the middle ear and the ear drum, the sensorineural hearing loss is related to a defect in the sensory hair cells in the cochlea [10]. The conductive hearing loss could in OI be related to fractures of the small bones or due to the changes in the collagen type 1 found in the ear drum itself. Whereas the sensorineural hearing loss may, in OI, be related to altered bone structure of the cochlea due to the altered collagen type 1 seen in OI which can affect the structure and function of the sensory hair cells [11].

A common complication in OI is early adult-onset hearing loss, though, the relationship between hearing loss and OI sub-type is still undetermined [8, 12, 13]. However, the hearing loss tends to be bilateral and progressive [14]. Mixed hearing loss is found in most age groups in people living with OI [12, 15, 16]. However, younger persons with OI tend to have conductive hearing loss [15], and older persons with OI tend to have sensorineural hearing loss [12, 14]. The exact prevalence reported by studies varies due to different definitions of hearing loss. A Danish study from 1984 by Pedersen et al. [17], including 201 people, found a prevalence of hearing loss of 50%, while a literature review by Carré et al. [12] found a prevalence of hearing loss ranging between 2 and 94%. Hearing loss in OI is multifactorial, the causes include fixation of the stapes footplate, thickened stapes footplate, atrophy of the stapes crura, microfractures, otosclerotic lesions, and/or obliteration of the round window [12, 14–18]. Conductive hearing loss in OI is usually treated with hearing aids or surgery, such as stapedotomy or stapedectomy, or in some severe cases of sensorineural hearing loss a cochlear implant [3].

Information about hearing loss, the temporal relationship, and treatment forms, is important for both patients and healthcare workers in managing hearing loss as a complication to osteogenesis imperfecta. Where previous studies have been cross-sectional studies limited by a low number of participants and the risk of selection bias, the present work is a population-based study with a long term follow-up due to the nature of the Danish Health Registers, which minimises the risk of these biases.

The aim of this study was to determine the prevalence, age at debut, incidence, and risk of hearing loss and the treatment thereof in the Danish OI Cohort. We hypothesised that hearing loss is more common among persons with OI, occurs at an earlier age, and that a higher proportion of people with OI need surgery or hearing aids due to OI-related changes in the bones of the middle ear.

## Method

This is a nationwide, register-based cohort study following persons with OI and a reference population from the 1st of January 1995 until date of death, date of emigration from Denmark, or the end of follow-up on the 31st of December 2018. We started the follow-up on the 1st of January 1995 as this marks the changes from World Health Organisation (WHO) International Classification of Diseases (ICD) either from the 8th edition (ICD-8) to 10th edition (ICD-10) in the Danish health registers. Furthermore, data on procedural codes were included at this date, allowing for the best capture of the endpoints included in this study (See below) [19].

### Outcomes, Variables, and Registers

Supplement Table 1 shows the included variables, registers, and corresponding codes used in this study. The date of the diagnosis for our end points was defined as the first entry into any of the included registers for the relevant event. The individual’s age for each event was calculated on the date of the registration. We defined hearing loss on an individual level as any registrations, in the included registers, with a diagnostic code related to hearing loss. This includes a diagnosis with conductive, sensorineural, or mixed hearing loss, or an otosclerosis diagnosis, or any procedural codes related to initiating hearing aids or cochlear implants, or any surgery on the bones of the middle ear. Similar methods have been used in other studies [20]. Otosclerosis was defined as any registrations of an otosclerosis diagnosis and/or a procedure code related to surgery on the middle ear. Surgery procedure codes for otosclerosis were included to increase capture, as treatment choices may have changed over time and are made in agreement with the patient and may include starting with hearing aids and subsequent surgery or opt for surgery as the first treatment. Hearing aids were defined as registration of any procedural codes related to hearing aids or cochlear implants.

**Table 1:** Participant characteristics

	OI cohort	Reference population	*p*-Value
Number of participants [*N*]	864	4276	NA
Female participants [*N* (%)]	487 (54.6%)	2330 (54.5%)	NA
Median age when entering registers in years [IQR]	0 [0–12]	0 [0–11.5]	NA
Median age at end observation in years [IQR]	32 [15–52]	30 [15–53]	NA
Median follow-up time per person in years [IQR]	28.6 [15.5–41.2]	27.8 [15.5–41.2]	NA
Median audiometries in hospital [N [IQR]]	0 [0–0]	0 [0–0]	< 0.001^w^
Total sum of audiometries in hospital [N]	1891	1175	NA
*Charlson comorbidity index*			< 0.001^c^
CCI = 0 [*N* (%)]	596 (69.0)	3491 (81.6)	
CCI = 1 [*N* (%)]	127 (14.7)	362 (8.5)	
CCI = 2 [*N* (%)]	56 (6.5)	182 (4.3)	
CCI ≥ 3 [*N* (%)]	85 (9.8)	241 (5.6)	

Participant basic characteristics for the OI cohort and reference population

*IQR* interquartile range, *CCI* Charlson comorbidity index, *w* Wilcoxon Signed Rank test, *c* Chi-squared-test

We calculated Charlson comorbidity index at the end of observation using the methods described by Christensen et al. [21] for all participants.

### Exposure and Observation Time

**Fig. 1:**
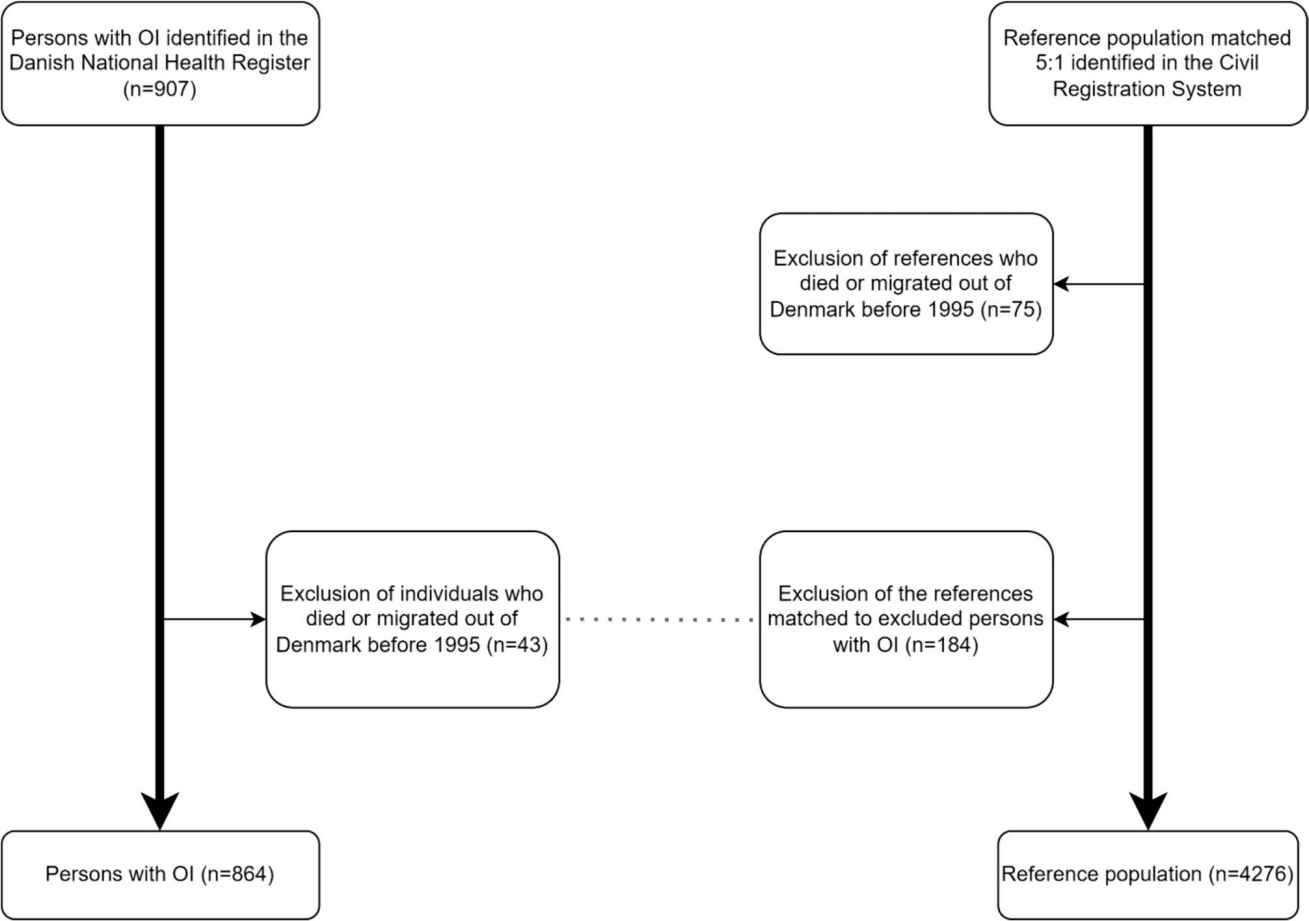
Inclusion of participants. Flow chart with inclusion and exclusion criteria for the OI cohort and reference population

Participants were included in the study on January 1st 1995, or at birth if born after January 1st 1995, or at the date of immigration to Denmark. All participants were followed until the end of observation, date of migration, or death. Figure 1 illustrates the inclusion and exclusion of participants.

### Data Source

The Civil Registration System (CRS) was introduced in 1968 in Denmark and includes a unique identification number known as the CRS number or CPR-number [19, 22]. The CRS registers name, gender, date and place of birth, date of death, citizenship, residential address, marital status, and migration in and out of Denmark [19]. The CPR-number can be used to link individuals and data between registers [22].

We used the National Patient register (NPR) in Denmark, which dates back to 1977 [19], to obtain data for this study. The NPR has health data registered from when an individual visits a public hospital and receives treatment or examination [23]. Diagnoses are recorded using the ICD-8 before 1994, and after that ICD-10 [19]. The ICD 9 was never used in Denmark [19]. Procedures are coded using the Danish Medical Classification System [23].

### Study Participants

The study cohort has been previously described in detail by Lyster et al. [7], but is summarized below. The OI cohort included every person registered in the NPR from January 1st 1977 until end of observation on December 31st 2018, with a WHO ICD-8 (756.59) or ICD-10 (Q78.0) diagnosis for OI. The reference population was matched so that there would be five persons for every one person with OI. Participants were randomly selected through the CRS, and matched on sex, birth month and -year. Persons with OI were excluded from the reference population, and neither could first- or second-degree relatives of persons with OI be included. Individuals were excluded if they died or migrated out of Denmark before 1995. All reference individuals had to be alive on the date the person with OI, to whom they were matched, received their diagnosis.

### Statistical Analysis

All statistical analyses were made using Stata 16.1 (StataCorp, USA).

Data are presented as mean ± SD or median [Interquartile Range], number of events and percent of the population, and events per 1000 person years as appropriate. Kaplan–Meier plots were created to visually assess event-free survival in both cohorts. Sub-hazard ratios (SHR) are presented as SHR between the OI cohort and the reference population, accepting a statistical significance if the 95% confidence interval (CI) of the SHR did not include the value 1.00. A Wilcoxon Signed Rank test was used to evaluate the between group differences in the number of audiometries and a chi-squared test to evaluate the distribution of Charlson Comorbidity Index (CCI) between the persons with OI and the reference population. A p-value of < 0.05 was accepted as indication of a statistically significant difference between the cohorts.

It has previously been shown that persons with OI have a shorter median lifespan compared to the reference population [1]. As the prevalence of hearing loss increases with age [10], it is likely that this competing risk will result in a lower incidence of hearing loss in the OI cohort. To evaluate the between group SHR we fitted a Fine and Gray [24] semi-parametic competing risk regression model for each outcome to take the competing risk of death into account, but did not adjust our models for other risk factors for hearing loss as many of these, such as noise exposure, are unavailable in the Danish registers. We tested if the proportional hazards assumption of the model held by visually assessing a log–log plot of survival, and accepting proportionality if the group graphs did not cross.

### Ethics

This study was approved by Statistics Denmark (jr.nr. 704542) all data were anonymised and depersonalised by Statistics Denmark. To preserve participant confidentiality, results are not shown when the total number of events was fewer than five.

## Results

907 persons with OI were identified and matched with 4535 in the reference population. Individuals who died or migrated out of Denmark before 1995 were excluded, leaving the study with 864 persons in the OI cohort (487 women), and 4276 persons (2330 women) in the reference population (Fig. 1). The participant characteristics are summarized in Table 1. The median follow-up time per person was 28.6 years (range 15.5–41.2) for the OI cohort and 27.8 (range 15.5–41.2) years for the reference population. The median age at entry into the OI cohort was 0 years (range 0–12 years), and 0 years (range 0–11.5 years) for the reference population. The median number of audiometries performed at the hospital was zero in both groups, but the 90th percentile was 4 or more audiometries per person in the OI cohort indicating a higher number of audiometries in the OI cohort. Persons with OI had more comorbidities than the reference population, as the CCI-score for the OI cohort was higher compared with the reference population (Table 1).

### Hearing Loss

**Fig. 2:**
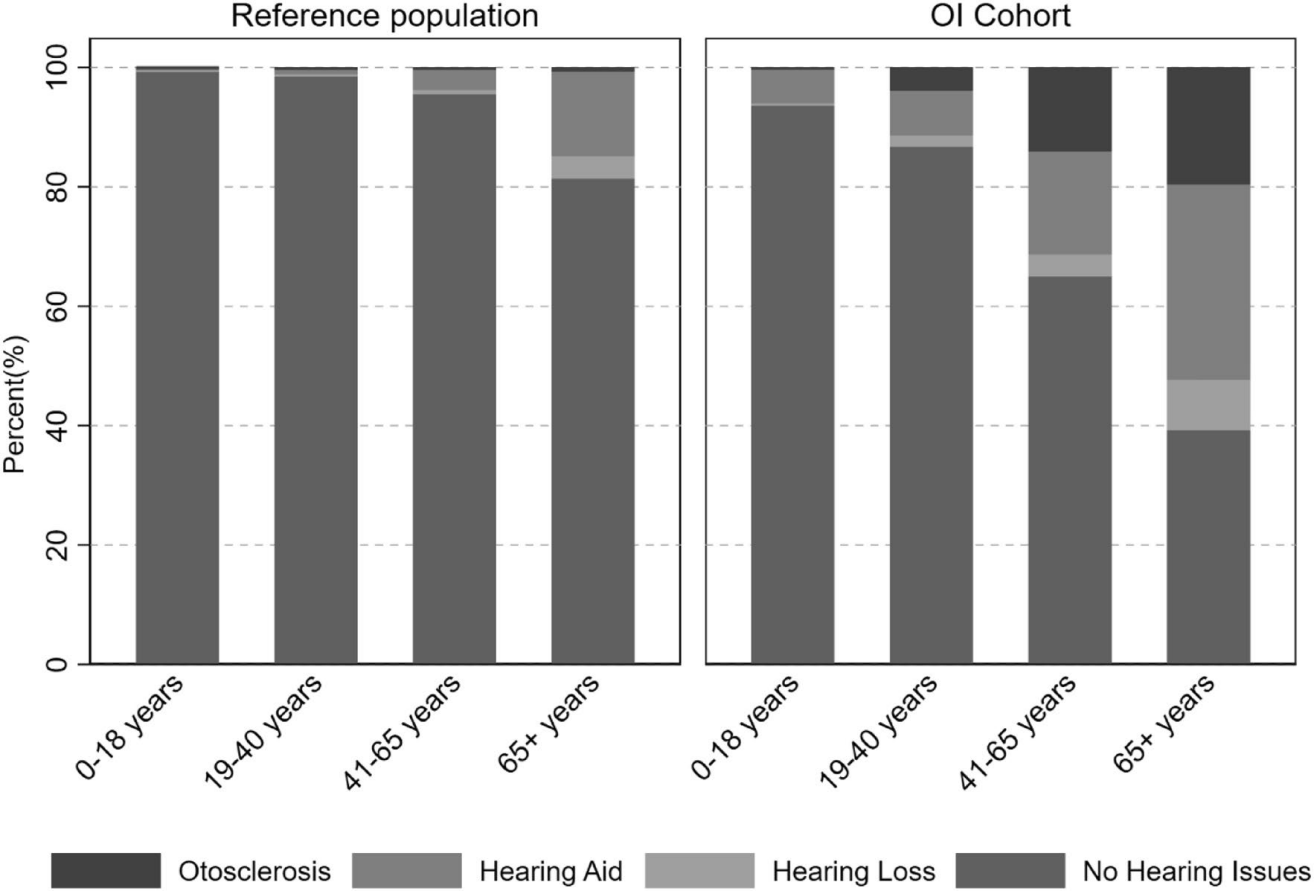
Age specific distribution of hearing loss in Osteogenesis Imeprfecta and the Reference Population. The figure. shows the percentage of participants with otosclerosis, hearing aid, unaided hearing loss (without hearing aid or otosclerosis), or no hearing loss-related diagnosis in each age group for the OI cohort and reference population

**Fig. 3:**
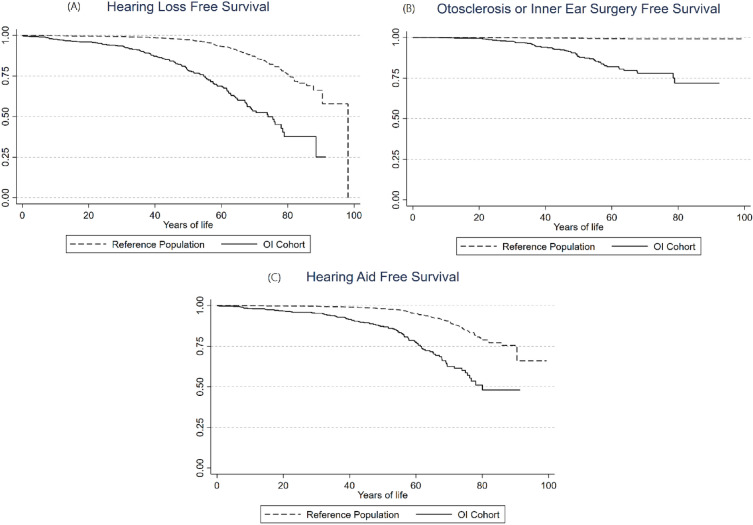
Kaplan Meier plots of hearing loss free survival. The figure shows the Kaplan–Meier curves of **A** any hearing loss free survival, **B** otosclerosis (including surgery to the middle ear and/or otosclerosis) free survival and **C** hearing aid free survival for the OI Cohort in solid lines and reference population in dashed lines

We found that 17.0% of the OI cohort and 4.0% of the reference population were diagnosed with any type of hearing loss during observation (Table 2). There was a lack of data concerning hearing loss subtype, but in the OI cohort 3.0% were registered with a conductive hearing loss diagnosis, 3.1% had a sensorineural hearing loss diagnosis, and 2.6% had a mixed hearing loss diagnosis. This corresponded to 0.3, 0.7, and 0.3%, respectively, in the reference population. The incident rate of any hearing loss was 5.15 per 1000 person years in the OI cohort, and 1.17 per 1000 person years in the reference population (SHR 4.56 [95% CI 3.64–5.71]) (Table 2). The median age for the hearing loss to occur was 42 years and 58 years for the OI cohort and reference population, respectively. Hearing loss increases in both cohorts with age, as shown in Fig. 2, but the percentage was greater in each age-bracket for the OI cohort. Figure 3A shows the Kaplan–Meier curve for hearing loss free survival, and shows that at approximately 75 years of age, half of the OI cohort are likely to have been diagnosed with hearing loss or have been treated for it.

**Table 2:** Risk of hearing loss in the OI Cohort compared to the reference population

Outcome	OI cohort			Reference population			Relative risk estimates
	*N* (%)	Median age at event in years [IQR]	IR n/1000 (95% CI)	*N* (%)	Median Age at Event in Years [IQR]	IR n/1000 (95% CI)	Sub-hazard ratio [95% CI]
Hearing loss defined as: any registrations, in the included registers, with a diagnostic code related to hearing loss. This includes conductive, sensorineural, and mixed hearing loss, *or* presence of otosclerosis, *or* any procedural codes related to initiating hearing aids or cochlear implants, *or* any surgery on the bones of the middle ear							
Total, hearing loss (any)	147 (17.0%)	42 [24–67]	5.15 (4.39–6.06)	172 (4.0%)	58 [43–69]	1.17 (1.00–1.35)	4.56 [3.64–5.71]
Men, hearing loss (any)	60 (15.3%)	34 [15–51]	5.08 (3.94–6.54)	56 (2.9%)	58 [39–68]	0.91 (0.70–1.18)	5.58 [3.86–8.06]
Women, hearing loss (any)	87 (18.4%)	46 [31–61]	5.21 (4.22–6.42)	116 (5.0%)	58 [45–70]	1.35 (1.13–1.62)	4.09 [3.07–5.44]
Otosclerosis and/or surgery defined as: any registrations of an otosclerosis diagnosis and/or a procedure code related to surgery on the middle ear							
Total, otosclerosis and/or surgery	63 (7.3%)	43 [31–55]	2.14 (1.67–2.74)	14 (0.3%)	32 [23–51]	0.09 (0.06–0.16)	22.51 [12.62–40.14]
Men, otosclerosis and/or surgery	26 (6.6%)	39 [28–50]	2.13 (1.45–3.13)	TFE	TFE	TFE	63.52 [15.07–267.78]
Women, Otosclerosis and/or Surgery	37 (7.8%)	46 [36–55]	2.15 (1.56–2.97)	TFE	TFE	TFE	15.60 [8.13–29.91]
Hearing aid defined as: registration of any procedural codes related to hearing aids or cochlear implants							
Total, hearing aid	108 (12.5%)	45 [22–60]	3.68 (3.05–4.45)	130 (3.0%)	60 [48–71]	0.88 (0.74–1.04)	4.16 [3.21–5.40]
Men, hearing aid	44 (11.2%)	36 [15–55]	3.62 (2.70–4.87)	40 (2.1%)	58 [43–67]	0.65 (0.48–0.88)	5.39 [3.50–8.31]
Women, hearing aid	64 (13.6%)	52 [32–62]	3.73 (2.92–4.76)	90 (3.7%)	62 [51–71]	1.04 (0.85–1.28)	3.63 [2.61–5.04]

The table shows the sum, prevalence, median age at event in years, incidence rate, sub-hazard ratio, and confidence interval of hearing loss, otosclerosis and/or surgery, and hearing aids for the OI and reference population, divided into total, men, and women

*IQR* interquartile range, *CI* confidence interval, *IR* incidence rate, *TFE* too few events

### Otosclerosis

7.3% of the people in the OI cohort had otosclerosis, while it was only 0.3% of the reference population. The median age for the event in the OI cohort was 43 years (range 31–55), and 32 years (range 23–51) in the reference group. Figure 2 shows that otosclerosis and/or surgery becomes progressively more common the higher the age-bracket for the OI cohort. Figure 3B shows that at approximately 80 years of age, 25% of the OI cohort are likely to have been diagnosed with or surgically treated for otosclerosis. The incidence rate was 2.14 per 1000 person years in the OI cohort and 0.09 per 1000 person years in the reference cohort (SHR 22.51 [95% CI 12.62–40.14]) (Table 2).

### Hearing Aids

In the OI cohort 12.5% had hearing aids, and in the reference population it was 3.0% (Table 2). Overall median age for getting a hearing aid was 45 years (range 22–60) in the OI cohort and 60 years (range 48–71) in the reference population (Table 2). There was a large difference in median age for men and women, ages being, respectively, 36 years (range 15–55) and 52 years (range 32–62). This difference was not as great in the reference group with median age for men being 58 years (range 43–67) and 62 years (range 51–71) for women. Figure 2 shows that use of hearing aids become more common in both the OI cohort and reference population in the higher age groups, but that this increase is greater for persons with OI. Figure 3C shows that 50% in the OI cohort were likely to use hearings aids by approximately 80 years old. The incidence rate for use of hearing aids was higher in the OI cohort than in the reference population (3.68 per 1000 person years vs 0.88 per 1000 person years, SHR 4.16 [95% CI 3.21–5.40]) (Table 2).

## Discussion

This study was a nationwide, register-based cohort study that included all persons registered with an OI diagnosis in Denmark matched to a reference population. We found a 17% prevalence of hearing loss amongst persons with OI, which was higher than that of the reference population, at 4%. Persons with OI had an overall higher risk of hearing loss with an earlier onset, already in the adolescent years. Furthermore, persons with OI were at higher risk of having otosclerosis and needing middle ear surgery therefore, though the median age was older than that of the reference population. People with OI were also more likely to use a hearing aid, and to use it at an earlier age, than the reference population. The prevalence for each of these events increased with age in the OI cohort.

The prevalence of hearing loss in our study, was lower than what have been found in other cross-sectional studies. In a North American study from 2020 including 312 individuals with OI by Machol et al. found a prevalence of hearing loss of 28% [25]. Whereas a study from 1984 including 201 people with OI by Pedersen et al. [17] found a prevalence of 50%. In a more resent Danish cross-sectional study including 85 adult persons with OI by Hald et al. [8], 63% of the participants evaluated had some type of hearing loss. In all of these studies the hearing loss diagnosis was based on performed audiometries, while our study is based on registered clinical diagnosis and procedural codes. Our results show an increase of prevalence of hearing loss or treatment thereof with age. This may explain the overall lower prevalence found in our study as our cohorts were younger, with a median age at the end of observation in the early thirties for both the OI cohort and reference population. Furthermore, our study included the total Danish OI Cohort, and is therefore less prone to selection bias of the study participants. We cannot, however, rule out that using our approach will under estimate the actual prevalence of hearing loss in the OI Cohort, as Hald et al. found that 20% of their participants with hearing loss and in need of intervention, were untreated and would not feature in the Danish Health Registers [8]. Our data may reflect the burden of hearing loss posed to the healthcare system and not necessarily that experienced by the people living with OI.

In our study the median age of being registered with some type of hearing loss was 42 years. This falls in line with most other studies that show an onset of a progressive hearing loss in the second to fourth decade of life for persons with OI [12, 13, 15].

The median age for otosclerosis was 43 years of age with a prevalence of 7.3% in the OI cohort. In the reference population this was younger, at 32 years of age, however, only 0.3% of the reference population were registered with a relevant otosclerosis code. This could explain the younger median age in the reference population. The prevalence remains largely the same in the reference population over time, while the prevalence increases with age in the OI cohort. We included the otosclerosis diagnosis and the surgical code related to any surgery done on bones of the middle ear to increase the capture of otosclerosis in the registers. Therefore, the prevalence in our study may not match the demand or number of surgeries actually performed. Otosclerosis can be treated with both stapes surgery and/or hearing aids [26, 27]. Therefore, a person with otosclerosis and treated with hearing aids can feature in both the hearing aids, as well as the otosclerosis categories.

In our population men was given a hearing aid earlier than women with OI. This may be due to differences in sound exposure, as seen in other populations [28]. The median age for being registered with a procedure concerning hearing aids was 45 years for the OI cohort and a median of 60 years for the reference population. However, it is possible that persons with OI are more likely to get their hearing aids through the public healthcare system, rather than through private hearing aid dispensers than the reference population. As only hearing aids dispensed through a hospital will be featured in our data set, it is possible that some of the people in the unaided hearing loss category, may have been treated with hearing aids through private hearing aid dispensers. In Denmark, people experiencing hearing loss will be seen by Ear Nose and Throat (ENT) Surgeons specialised in hearing loss in the hospitals or via private practice ENT clinics. If hearing loss is diagnosed patients will be referred to either hospital or private clinics for hearing aid fitting. We cannot rule out that our findings are influenced by socio-economic factors, but we would argue that any bias would be similar between the two groups.

We acknowledge that there are several limitations with this study. First, private hearing aid dispensers are not covered in our data sources. As OI is a rare disease, and persons with OI are followed at a few centres of expertise, it is likely that this population will be seen in a hospital setting more often than the reference population who will be more likely to be followed in the primary health care system for diseases such as conductive hearing loss. In Denmark 60% of all hearing aids are dispensed through the public health care system. While this may skew the comparison of the two groups, the results for the frequency of events in the OI cohort should be representative. Secondly, we could not access the audiometries performed outside of a public hospital setting, for example, at private ENT doctors and hearing aid dispensers, where most of these are performed in the general population. Furthermore, we were not powered to evaluate changes in treatment modalities over time or incidence over time. Lastly, we have no information about the genetic background of the OI diagnosis nor the clinical severity of the diseases.

A strength of this study is the large study population gathered from the validated data sources with automated data capture with virtually no loss to follow-up or selection bias. This is a nationwide register for a universal tax financed healthcare system that is free to use for all inhabitants of Denmark. The NPR has 99% of all hospital contacts and 95% of all surgical procedures registered [19]. Furthermore, we know that the positive predictive value of having a disease that you are registered with in our data sources is above 95% [22]. Furthermore, the risk of not having OI if you are registered with an OI diagnosis is below 5% [29].

## Conclusion

We found that 17% of persons with OI are registered with some form of hearing loss and/or treated thereof at a much younger age than the general population. Furthermore, we found an increase in incidence of hearing loss with 50% of the OI population, by the age of 75 suffering from hearing loss.

Regular follow-up may be needed in the OI population, but further research is needed to evaluate the risk of hearing loss-related to genotype and clinical severity.
